# Supplementary material for: Metabolomics analysis of human acute graft-versus-host disease reveals changes in host and microbiota-derived metabolites
Source: Nat Commun. 2019 Dec 13;10:5695. doi: 10.1038/s41467-019-13498-3 (PMC6910937; doi:10.1038/s41467-019-13498-3)
Supplement: Supplementary file 1 — Supplementary Information [file 41467_2019_13498_MOESM1_ESM.pdf]

Supplementary information

Metabolomics analysis of human acute Graft-versus-Host Disease reveals changes in host and microbiota-derived metabolites

Michonneau et al.

Supplementary figure 1, Michonneau et al.

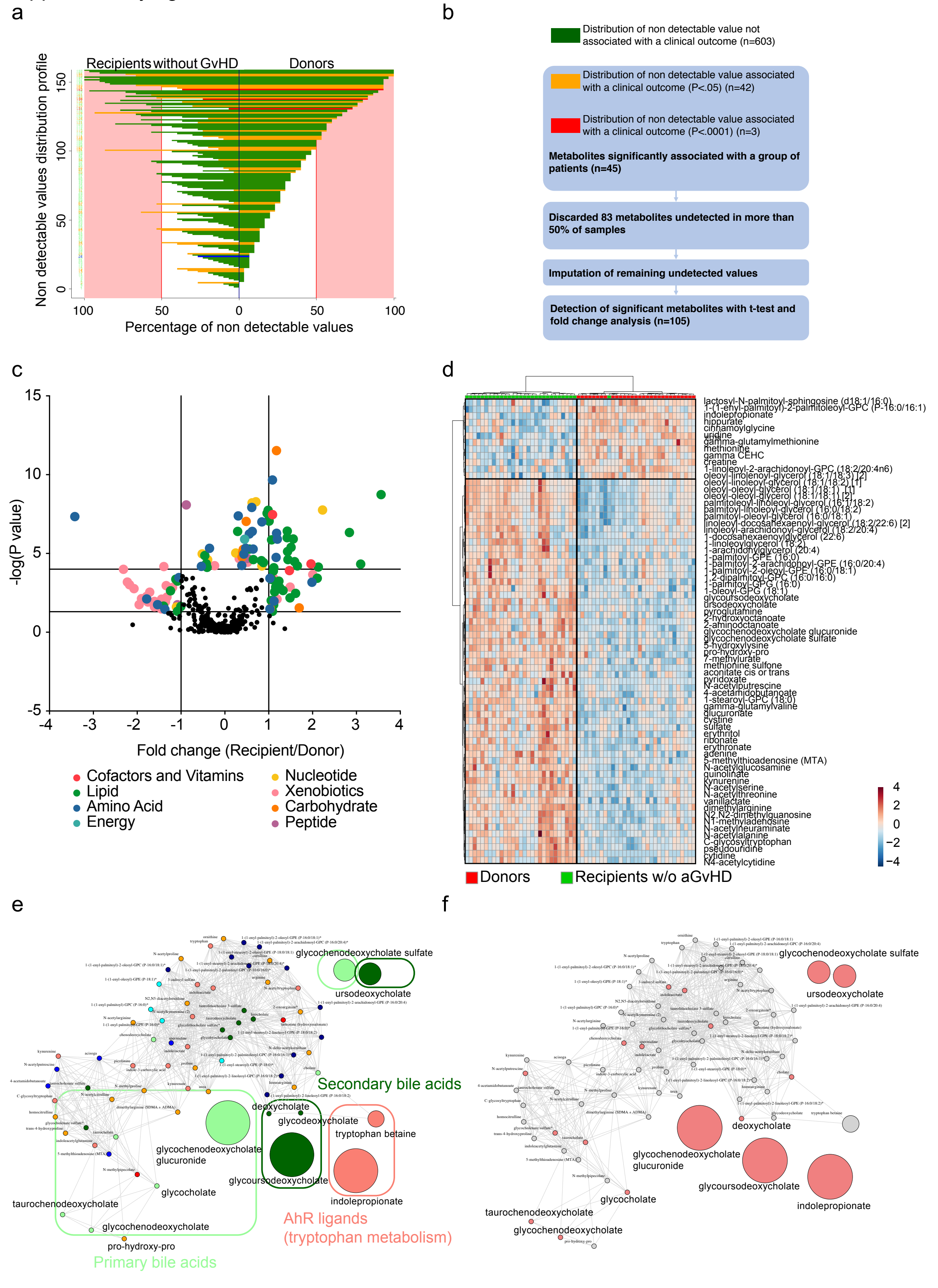

Supplementary figure 1: Metabolomics changes after transplantation in recipients without GvHD in the confirmation multicentric cohort

(a) Distribution of non-detectable values between recipient without GvHD and their related paired donors for the same 648 metabolites analyzed in cohort 1 (Figure 2). The frequency of non-detectable values in both groups

was compared using a McNemar exact test and colored as follow; green: frequency of non-detectable values non significantly different between groups, blue: combination of metabolites with $p>0.05$ or $p<0.05$ after paired analysis, orange: frequency of non-detectable values significantly different between groups with $p < 0.05$ but not significant after Bonferroni correction for multiple testing, red: frequency of non-detectable values significantly different between groups after Bonferroni correction for multiple testing ($p < 7.692\times10^{-5}$). Red area represents the threshold used for filtration of metabolites with more than 50 % of non-detectable value. **(b)** A total number of 648 metabolites were analyzed for comparison of paired donor and recipients without GvHD. Among them, 45 metabolites were more frequently detected in one group of patients, including 3 metabolites that were still significant after Bonferroni correction (supplementary data 3). 83 metabolites with more than 50% of non-detectable values were discarded for further analysis. **(c)** The remaining 565 metabolites were compared with a paired Student test followed by a Bonferroni correction. The volcano plot represents the variation of metabolites amount between recipients without GvHD and their related paired donors according to the −log(p value). List of the 105 significant metabolites is available in supplementary data 5. **(d)** Heatmap representation of the more significant metabolites after Student test, after hierarchical clustering of samples. **(e-f)** Significant variation of metabolites was confirmed after global comparison of the relative amounts between compounds. Main pathways identified in the previous analysis (i.e. polyamine metabolism, tryptophan metabolism, urea cycle, arginine & proline metabolism, bacterial or fungal, plasmalogen or lysoplasmalogen, and primary or secondary bile acid metabolism) were used to build an undirected graph where each node is a metabolite and two nodes are connected if their ratio is unchanged between the two groups (see Methods). The same network was first colored according to the considered sub-pathway **(e)** and then according to microbial-derived metabolites (red nodes) **(f)**.

Supplementary figure 2, Michonneau et al.

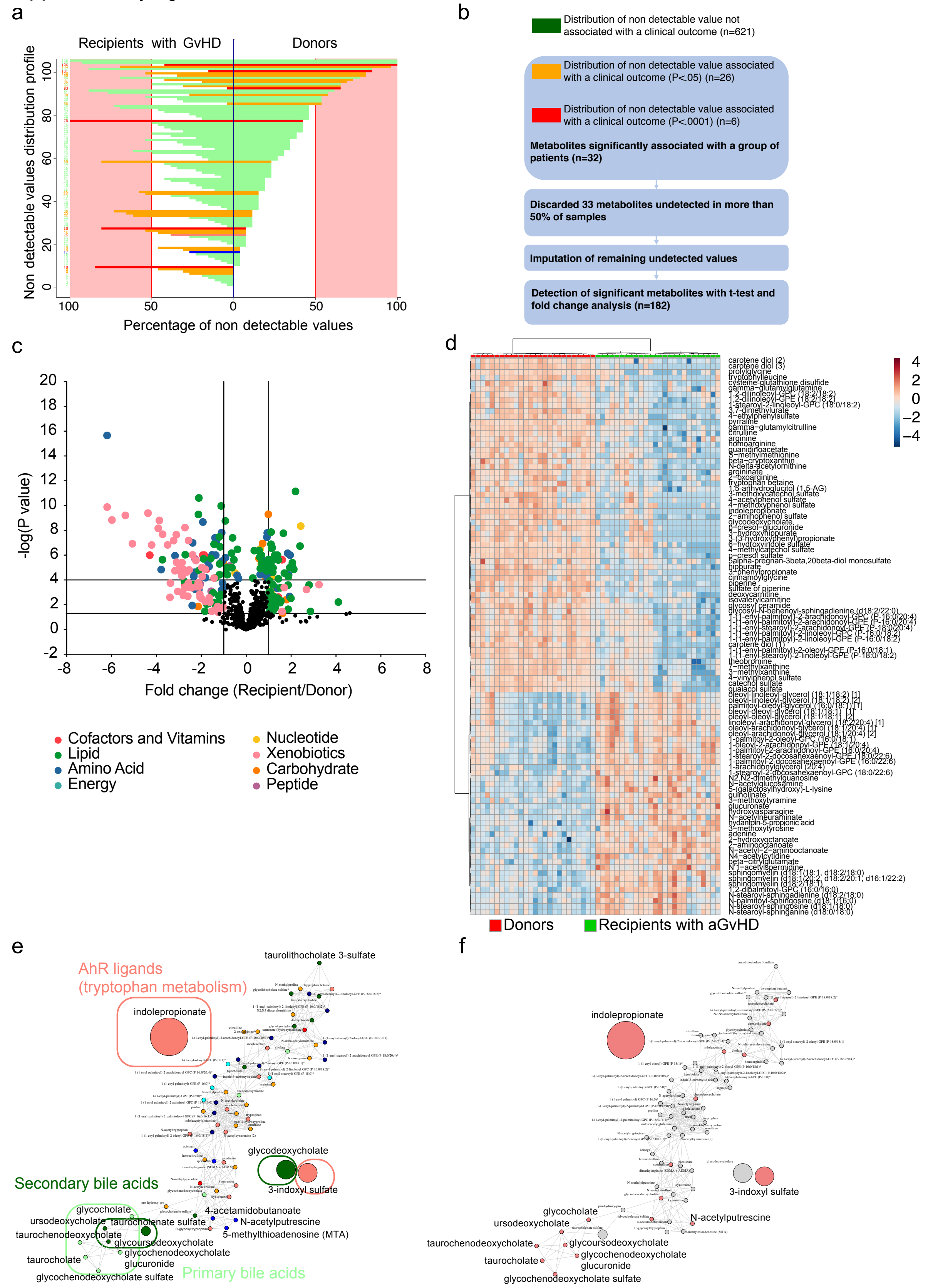

Supplementary Figure 2: Metabolomics changes after transplantation in recipients with GvHD in the multicentric confirmation cohort

(a) Distribution of non-detectable values for each metabolite between recipient without GvHD and their related paired donors for the same 653 metabolites analyzed in cohort 1 (Figure 3). The frequency of non-

detectable values in both groups was compared for each distribution profile using a McNemar exact test and colored as follow; green: frequency of non-detectable values non significantly different between groups, blue: combination of metabolites with $p>0.05$ or $p<0.05$ after paired analysis, orange: frequency of non-detectable values significantly different between groups with $p < 0.05$ but not significant after Bonferroni correction for multiple testing, red: frequency of non-detectable values significantly different between groups after Bonferroni correction for multiple testing ($p < 7.692\times10^{-5}$). Red area represents the threshold used for filtration of metabolites with more than 50 % of non-detectable value. **(b)** A total number of 653 metabolites were analyzed for comparison of paired donor and recipients without GvHD. Among them, 32 metabolites were more frequently detected in one group of patients, including 6 metabolites that were still significant after Bonferroni correction: cytidine, glycodeoxycholate, indolepropionate, L-urobilin, ursodeoxycholate sulfate and 3-methoxytyramine sulfate (supplementary data 6). 33 metabolites with more than 50% of non-detectable values were discarded for further analysis. **(c)** The remaining 620 metabolites were compared with a paired Student test followed by a Bonferroni correction. The volcano plot represents the variation of metabolites amount between recipients without GvHD and their related paired donors according to the −log(p value). List of the 182 significant metabolites is available in supplementary data 8. **(d)** Heatmap representation of the more significant metabolites after Student test, after hierarchical clustering of samples. **(e-f)** Significant variation of metabolites was confirmed after global comparison of the relative amounts between compounds. Main pathways identified in the previous analysis (i.e. polyamine metabolism, tryptophan metabolism, urea cycle, arginine & proline metabolism, bacterial or fungal, plasmalogen or lysoplasmalogen, and primary or secondary bile acid metabolism) were used to build an undirected graph where each node is a metabolite and two nodes are connected if their ratio is unchanged between the two groups (see Online Methods). The same network was first colored according to the considered sub-pathway **(e)** and then according to microbial-derived metabolites (red nodes) **(f)**.

Supplementary figure 3, Michonneau et al.

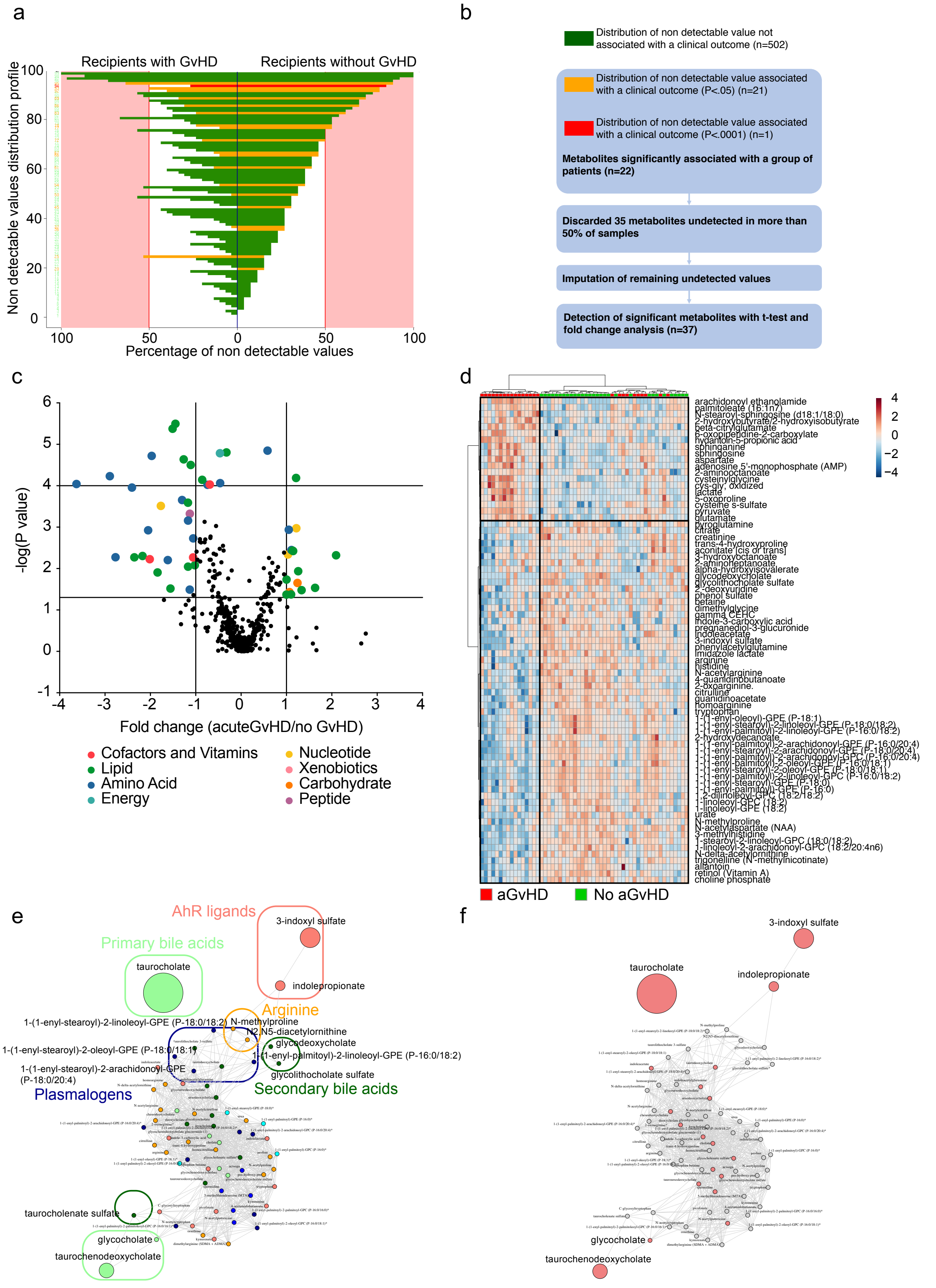

Supplementary figure 3: Metabolomics pathways involved at aGvHD onset in the multicentric confirmation cohort

(a) Distribution of non-detectable values for each of the 524 metabolites previously analyzed in cohort 1 (figure 4), was assessed in recipients with or without GvHD. The frequency of non-detectable values in both groups was

compared for each distribution profile using a Fisher exact test for 2x2 contingency table and colored as follow; green: frequency of non-detectable values non-significantly different between groups, orange: frequency of non-detectable values significantly different between groups with $p<0.05$ but not significant after Bonferroni correction for multiple testing, red: frequency of non-detectable values significantly different between groups after Bonferroni correction for multiple testing. **(b)** A total number of 524 metabolites were analyzed for comparison of recipients with or without GvHD. Among them, 22 metabolites were more frequently detected in one group of patients, and one was significant after Bonferroni correction (indoleproprionate, $p<0.0001$) (supplementary data 10). 35 metabolites with more than 50% of non-detectable values were discarded for further analysis. **(c)** The remaining 489 metabolites were compared with a Student test with unequal variance, followed by a Bonferroni correction after log transformation of peak intensities. The volcano plot represents the variation of metabolites amount between recipients with GvHD and those without GvHD according to the −log(p value). List of the 37 significant metabolites is available in supplementary data 12. **(d)** Heatmap representation of the more significant metabolites after Student test with unequal variance, after hierarchical clustering of samples. **(e-f)** Significant variation of metabolites was confirmed after global comparison of the relative amounts between compounds. Main pathways identified in the previous analysis (i.e. polyamine metabolism, tryptophan metabolism, urea cycle, arginine & proline metabolism, bacterial or fungal, plasmalogen or lysoplasmalogen, and primary or secondary bile acid metabolism) were used to build an undirected graph of a network where each node is a metabolite and two nodes are connected if their ratio is unchanged between the two groups (see Online Methods). The same network was first colored according to the considered sub-pathway **(e)** and then according to microbial-derived metabolites (red nodes) **(f)**.
