# Supplementary material for: Metabolomics analysis of human acute graft-versus-host disease reveals changes in host and microbiota-derived metabolites
Source: Nat Commun. 2019 Dec 13;10:5695. doi: 10.1038/s41467-019-13498-3 (PMC6910937; doi:10.1038/s41467-019-13498-3)
Supplement: Supplementary file 3 — Description of Additional Supplementary Files [file 41467_2019_13498_MOESM3_ESM.docx]

**Description of Supplementary Files**

**File Name:** Supplementary Data 1

**Description:** Patient's characteristics

**File Name:** Supplementary Data 2

**Description:** metabolites more frequently detected in donors or recipient without GvHD (cohort 1, Saint Louis)

**File Name:** Supplementary Data 3

**Description:** metabolites more frequently detected in donors or recipient without GvHD (cohort 2, Cryostem)

**File Name:** Supplementary Data 4

**Description:** list of metabolites significantly changing between donors and their paired recipients without GvHD (Cohort 1, Saint Louis)

**File Name:** Supplementary Data 5

**Description:** list of metabolites significantly changing between donors and their paired recipients without GvHD (Cohort 2, Cryostem)

**File Name:** Supplementary Data 6

**Description:** metabolites more frequently detected in donors or recipient with GvHD (cohort 2, Cryostem)

**File Name:** Supplementary Data 7

**Description:** list of metabolites significantly changing between donors and their paired recipients with GvHD (Cohort 1, Saint Louis)

**File Name:** Supplementary Data 8

**Description:** list of metabolites significantly changing between donors and their paired recipients with GvHD (Cohort 2, Cryostem)

**File Name:** Supplementary Data 9

**Description:** metabolites more frequently detected in recipient with or without GvHD (Cohort 1, Saint Louis)

**File Name:** Supplementary Data 10

**Description:** metabolites more frequently detected in recipient with or without GvHD (Cohort 2, Cryostem)

**File Name:** Supplementary Data 11

**Description:** list of metabolites significantly changing at GvHD onset (Cohort 1, Saint Louis)

**File Name:** Supplementary Data 12

**Description:** list of metabolites significantly changing at GvHD onset (Cohort 2, Cryostem)

**File Name:** Supplementary Data 13

**Description:** Metabolites associated with acute GvHD onset after adjustment for age, gender and BMI

**File Name:** Supplementary Data 14

**Description:** identification of metabolites associated with GvHD with Lasso logistic regression analysis and sparse Partial Least Square Discriminant Analysis.

**File Name:** Supplementary Data 15

**Description:** Limit of Detection (LOD) for Standards analyzed in a Dilution Series Using ReversePhase Chromatography

**File Name:** Supplementary Data 16

**Description:** list of total metabolites identified in both cohorts

**File Name:** Supplementary Data 17

**Description:** threshold used in the different datasets for undirected graph building
