# Supplementary material for: Metabolomics analysis of human acute graft-versus-host disease reveals changes in host and microbiota-derived metabolites
Source: Nat Commun. 2019 Dec 13;10:5695. doi: 10.1038/s41467-019-13498-3 (PMC6910937; doi:10.1038/s41467-019-13498-3)
Supplement: Supplementary file 21 — Reporting Summary [file 41467_2019_13498_MOESM21_ESM.pdf]

nature research

Corresponding author(s): Gérard Socié

Last updated by author(s): Nov 8, 2019

Reporting Summary

Nature Research wishes to improve the reproducibility of the work that we publish. This form provides structure for consistency and transparency in reporting. For further information on Nature Research policies, see Authors & Referees and the Editorial Policy Checklist .

Statistics

For all statistical analyses, confirm that the following items are present in the figure legend, table legend, main text, or Methods section.

n/a	Confirmed	
☐	☒	The exact sample size (*n*) for each experimental group/condition, given as a discrete number and unit of measurement
☐	☒	A statement on whether measurements were taken from distinct samples or whether the same sample was measured repeatedly
☐	☒	The statistical test(s) used AND whether they are one- or two-sided<br>*Only common tests should be described solely by name; describe more complex techniques in the Methods section.*
☐	☒	A description of all covariates tested
☐	☒	A description of any assumptions or corrections, such as tests of normality and adjustment for multiple comparisons
☐	☒	A full description of the statistical parameters including central tendency (e.g. means) or other basic estimates (e.g. regression coefficient) AND variation (e.g. standard deviation) or associated estimates of uncertainty (e.g. confidence intervals)
☐	☒	For null hypothesis testing, the test statistic (e.g. *F*, *t*, *r*) with confidence intervals, effect sizes, degrees of freedom and *P* value noted<br>*Give P values as exact values whenever suitable.*
☒	☐	For Bayesian analysis, information on the choice of priors and Markov chain Monte Carlo settings
☐	☒	For hierarchical and complex designs, identification of the appropriate level for tests and full reporting of outcomes
☒	☐	Estimates of effect sizes (e.g. Cohen's *d*, Pearson's *r*), indicating how they were calculated

Our web collection on statistics for biologists contains articles on many of the points above.

Software and code

Policy information about availability of computer code

Data collection	No software was used for data collection
Data analysis	All statistical analysis were performed with R v3.5.1 (https://www.r-project.org/). The following packages were used for data analysis : SARP Compo (https://cran.r-project.org/), glmnet (https://www.jstatsoft.org/article/view/v033i01), glmmlasso(https://cran.r-project.org/web/packages/glmmLasso/index.html), stats (https://cran.r-project.org/), plsRglm(https://cran.r-project.org/web/packages/plsRglm/index.html), FactoMineR (https://www.r-project.org/ and http://factominer.free.fr/index.html), factoextra (https://cran.r-project.org/web/packages/factoextra/index.html), mixOmics (https://www.r-project.org/), MetaboAnalystR (https://github.com/xia-lab/MetaboAnalystR).

For manuscripts utilizing custom algorithms or software that are central to the research but not yet described in published literature, software must be made available to editors/reviewers. We strongly encourage code deposition in a community repository (e.g. GitHub). See the Nature Research guidelines for submitting code & software for further information.

Data

Policy information about availability of data

All manuscripts must include a data availability statement. This statement should provide the following information, where applicable:

- Accession codes, unique identifiers, or web links for publicly available datasets
- A list of figures that have associated raw data
- A description of any restrictions on data availability

Raw metabolomic data that support the findings of this study have been deposited in MetaboLights repository with the following accession code: MTBLS204 (www.ebi.ac.uk/metabolights/mtbls204) (cohort 1) and MTBLS205 (https://www.ebi.ac.uk/metabolights/mtbls205) (cohort 2).

Field-specific reporting

Please select the one below that is the best fit for your research. If you are not sure, read the appropriate sections before making your selection.

- [x] Life sciences
- [ ] Behavioural & social sciences
- [ ] Ecological, evolutionary & environmental sciences

For a reference copy of the document with all sections, see nature.com/documents/nr-reporting-summary-flat.pdf

Life sciences study design

All studies must disclose on these points even when the disclosure is negative.

Sample size	No sample size was calculated. Inclusion criteria were adult patients (more than 18-year-old), with a sibling identical donor who underwent an allogeneic HSCT. Our objectives were to include at least 40 patients per cohort with an expected acute GvHD incidence of 40%.
Data exclusions	Patients with HIV or HTLV co-infection were excluded from the study. No acquired data was excluded from the analysis.
Replication	In order to replicate results obtained with the monocentric cohort from Saint Louis hospital (discovery cohort), a second multicentric cohort (replication cohort) was obtained from a national biobank (Cryostem).
Randomization	No randomization was required in this study.
Blinding	Investigators and technicians were blinded to patient graft-versus-host disease status and clinical outcomes at the time of sample processing. Statisticians that analyzed data did not contribute to experimental procedures.

Reporting for specific materials, systems and methods

We require information from authors about some types of materials, experimental systems and methods used in many studies. Here, indicate whether each material, system or method listed is relevant to your study. If you are not sure if a list item applies to your research, read the appropriate section before selecting a response.

Materials & experimental systems

n/a	Involved in the study	
x		Antibodies
x		Eukaryotic cell lines
x		Palaeontology
x		Animals and other organisms
	x	Human research participants
x		Clinical data

Methods

n/a	Involved in the study	
x		ChIP-seq
x		Flow cytometry
x		MRI-based neuroimaging

Human research participants

Policy information about studies involving human research participants

Population characteristics	Patients analyzed in this study were hospitalized for allogeneic hematopoietic stem cell transplant in the Adult Hematology and Transplantation unit of Saint-Louis hospital, Paris, France (cohort 1) or in one of the 33 transplantations center from Cryostem consortium (cohort 2). Inclusion criteria were adult patients (18 years old or older) who underwent a sibling-identical allogeneic hematopoietic stem cell transplantation. Clinical data extracted from patient medical records included age, gender, diagnosis, HLA matching between donor and recipient, conditioning regimen, T-cell depletion, presence or absence of GVHD, and graft characteristics. In patients with GVHD, the location, grade, chronicity, and treatment were recorded. If relapse or death occurred, the dates and cause of death were also recorded.
Recruitment	All patients who underwent a sibling-identical allogeneic hematopoietic stem cell transplantation between November 1st 2012 and June 1st 2015 for the monocentric cohort from Saint Louis hospital.<br>All patients with sibling-identical allogeneic HSCT, more than 18 years old, with available samples for recipients and for their related donors, transplanted between May 1st 2013 and July 1st 2016, were collected for the multicentric cohort from Cryostem biobank.
Ethics oversight	All patients gave their written consent for clinical research. This non-interventional research study with no additional clinical procedure was carried out in accordance with the Declaration of Helsinki. Data analyses were carried out using a database with all patient identifiers removed. This study was declared to the CNIL (Commission National Informatique et Liberté, number KoT1175225K) and was approved by the local ethic committee and Institutional Review Board (CPP Ile de France IV, IRB number 00003835).

Note that full information on the approval of the study protocol must also be provided in the manuscript.
